# Piezoelectric Ultrasonic Biological Microdissection Device Based on a Novel Flexure Mechanism for Suppressing Vibration

**DOI:** 10.3390/mi12020196

**Published:** 2021-02-13

**Authors:** Haibo Huang, Yifan Pan, Yan Pang, Hao Shen, Xiwei Gao, Yichen Zhu, Liguo Chen, Lining Sun

**Affiliations:** 1 Jiangsu Provincial Key Laboratory of Advanced Robotics, School of Mechanical and Electric Engineering, Soochow University, Suzhou 215123, China; hbhuang@suda.edu.cn (H.H.); yfpannes@stu.suda.edu.cn (Y.P.); 20205229040@stu.suda.edu.cn (Y.P.); 20194029003@stu.suda.edu.cn (H.S.); XiweiGAO@hotmail.com (X.G.); lnsun@hit.edu.cn (L.S.); 2 Cambridge-Suda Genomic Resource Center, Soochow University, Suzhou 215123, China; 3 State Key Laboratory of Robotics & Systems, Harbin Institute of Technology, Harbin 150001, China

**Keywords:** microdissection, ultrasonic vibration, flexure mechanism, tissue section

## Abstract

Biological microdissection has a wide range of applications in the field of molecular pathology. The current laser-assisted dissection technology is expensive. As an economical microdissection method, piezoelectric ultrasonic microdissection has broad application prospects. However, the performance of the current piezoelectric ultrasonic microdissection technology is unsatisfactory. This paper aims to solve the problems of the low dissecting precision and excessive wear of the dissecting needle caused by the harmful lateral vibration of the present piezoelectric ultrasonic microdissection device. A piezoelectric ultrasonic microdissection device based on a novel flexure mechanism is proposed. By analyzing the flexure hinge flexibility, the type of flexure beam and the optimal design parameters are determined. Through harmonic response simulation analysis, the newly designed microdissection device with a vibration-suppressing mechanism achieves the best vibration effect when the driving frequency is 28 kHz. Under this driving frequency, the lateral vibration suppression effect is improved by 68% compared to the traditional effect without vibration suppression. Then, based on 3D printing technology, a prototype of a novel microdissection device is produced, and its performance is tested. Experiments on dissecting needle vibration tests show that the flexure mechanism does indeed suppress the lateral vibration of the needle tip. We conducted various tissue dissection experiments on paraffin tissue sections. First, we determine the optimal dissecting parameters (driving voltage, frequency, feed speed, cutting angle) of the new equipment through various parameter dissecting experiments. Then, we adopt these optimal dissecting parameters to perform three kinds of dissecting experiments on mouse tissue paraffin section (liver, lung, bone), dissecting experiments on tissue sections of different thicknesses (3 μm, 4 μm, 5 μm), sampling and extraction experiments on complete tissue. The new device has a better dissecting performance for paraffin tissue sections below a 5 μm thickness and can complete various dissecting tasks. Finally, we compare the wear of the dissecting needles of the new and old devices after the same dissecting tasks. The results prove that the suppression of harmful lateral vibration not only significantly improves the dissecting effect but also increases the service life and durability of the dissecting needle, which is beneficial for reducing the equipment costs.

## 1. Introduction

Microdissection technology refers to the selective acquisition of single cells or cell groups from biological tissue slices through micromanipulation systems, separating them from surrounding tissues, and collecting the separated cells for subsequent analysis [1,2]. Microdissection has been widely used in the analysis of tumor molecular heterogeneity [3], cell mutation [4], and chromosome structure analysis [5]. With the continuous development of technology, a variety of different methods have emerged for microdissection, including direct manual microdissection [6], laser-capture microdissection [7,8,9], and piezoelectric ultrasonic dissection [10,11,12,13,14].

Direct manual microdissection has low precision [15], poor repeatability, and high technical requirements for operators, so it is only suitable for large-area dissection. Laser-capture microdissection technology can quickly capture more target tissues from many research materials, and it is currently the most widely used dissecting method [16]. Laser-capture microdissection has been widely used to analyze pathological tissues with its advantages of high precision and rapid tissue separation. However, this technique requires special equipment, which is expensive and may potentially cause contamination from laser radiation during cell capture and separation [17].

Compared with laser dissection technology, piezoelectric ultrasonic microdissection technology uses a piezoelectric actuator to drive the dissecting needle to perform high-frequency vibration [18]. Therefore, regardless of the direction you dissect, the needle tip is similar to a sharp saw. It can cleanly separate the cells in the target area without contaminating the sample tissue due to laser radiation and infrared heating. Besides, piezo-power microdissection (PPMD) technology does not require expensive special equipment [19] and can be afforded by ordinary laboratories.

However, the current PPMD dissection device has some shortcomings. During the working process of the dissection device, piezoelectric ceramics will not only generate the axial vibration required for dissection but also generate lateral vibration, which is harmful to the dissection. Excessive lateral vibration results in uneven dissecting edges and significantly reduces dissecting precision. It also accelerates the wear of the dissecting needle, making the dissection ineffective. Therefore, effectively suppressing the lateral vibration is the key to further improving the dissecting effect of PPMD.

In this paper, aiming at these problems, a piezoelectric ultrasonic microdissection device based on a novel flexure mechanism is proposed. The novel flexure mechanism can effectively filter and absorb the vibration energy generated by a piezoelectric actuator, retain the axial vibration that is beneficial to dissection, and suppress the lateral vibration, which is harmful to dissection. In Section two, the type of flexure beam and the optimal design parameters of the flexure mechanism are determined by theoretical analysis. In Section three, the harmonic response comparative analysis was carried out with the traditional piezoelectric microdissection device without an integrated flexure vibration damping structure using finite element simulation analysis. The simulation results show that the dissection device design presented in this paper has the best vibration effect at 28 kHz, and its spatial lateral amplitude evaluation value is 1.59 μm. In contrast, the traditional dissection device has an optimal spatial lateral amplitude evaluation value of 5.09 μm. The lateral amplitude suppression effect increased by 68%. In Section 4, based on 3D printing technology, a prototype of the new type of microdissection device was made, and its performance was tested. Experimental results show that the dissection device based on the flexure mechanism had no evident lateral vibration on the tip under the optimal vibration frequency, the flexure mechanism did indeed suppress the needle tip’s lateral vibration. We conducted various tissue dissecting experiments. First, through repeated experiments and comparisons, the optimal dissecting parameters are obtained. When the dissection device works at 28 kHz, the driving voltage is 12 V, the feed speed is 100 μm/s, and the dissecting angle is 45°, the optimal dissecting effect can be obtained. Then, we adopted these optimal dissecting parameters to perform three kinds of mouse tissue paraffin sections (liver, lung, bone) dissecting experiments, different thickness tissue sections (3 μm, 4 μm, 5 μm) dissecting experiments, as well as complete tissue sampling and extraction experiments. The new device can accomplish various tasks well. Finally, we compared the wear of the dissecting needles of the new and old devices after the same dissecting tasks. The results can prove that the suppression of harmful lateral vibration not only significantly improves the dissecting effect but also increases the service life and durability of the dissecting needle, which is beneficial to reduce the equipment costs.

## 2. Structural Design

The microdissection needle is connected to the piezoelectric actuator with one end fixed and the other free. In the microdissection process, the piezoelectric actuator’s energy is transferred to the needle tip over a distance. However, due to specific errors in processing and assembly, the piezoelectric actuator not only outputs the axial displacement for microdissection but also produces lateral displacement that is harmful to dissection. These harmful lateral displacements are amplified at the tip of the needle over a certain distance. Excessive lateral vibration easily blurs the dissecting edge during the dissecting process, which leads to an unsatisfactory dissecting effect. Meanwhile, a collision between the tip and the slice base is inevitable, accelerating the dissecting needle tip’s wear and making the dissection ineffective. Therefore, suppressing the lateral output energy of piezoelectric ceramics is the key to reducing lateral vibration.

### 2.1. The Overall Design of the Microdissection Device

**Figure 1:**
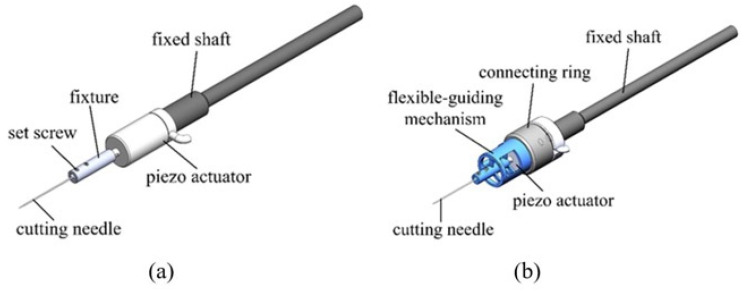
Two piezoelectric ultrasonic microdissection devices: (**a**) Piezoelectric microdissection device with traditional structure; (**b**) piezoelectric microdissection device based on flexure mechanism.

Figure 1a shows a schematic diagram of a traditional microdissection device. The traditional piezoelectric microdissection device consists of a fixed rod, a piezoelectric actuator, a clamp, a set screw, and a dissecting needle. During the dissecting process, the piezoelectric actuator’s energy directly acts on the microdissection needle, and the energy of the lateral vibration is amplified as the transmission distance increases. Therefore, this structure cannot achieve precise tissue slices and is only suitable for larger-scale dissection.

A flexure mechanism is introduced in the design. Figure 1b illustrates the schematic diagram of a new ultrasonic piezoelectric microdissection device based on a flexural mechanism. This new design comprises a fixed rod, a connecting base, an outer shell, a packaged piezoelectric ceramic, a flexure mechanism, and a dissecting needle. To facilitate the assembly, the piezoelectric ceramic p-855.11 (5 mm × 5 mm × 9 mm) of the PI company was selected and encapsulated in a stainless steel case with 200 N pretension applied. The energy generated by the piezoelectric ceramics is filtered and absorbed by the flexure mechanism and transferred to the dissecting needle to obtain a small axial vibration, thereby improving the precision of microdissection.

### 2.2. Design and Analysis of Flexure Hinge

The flexure mechanism is a device that relies on the elastic deformation of the flexure hinge itself to realize movement, force, or energy transfer and conversion [20]. Compared with the traditional rigid mechanism, it has many advantages, including no gap, no friction, small size, lightweight, integrated design and processing, no assembly, and high precision. It is widely applied in precision positioning and other fields [21,22].

**Figure 2:**
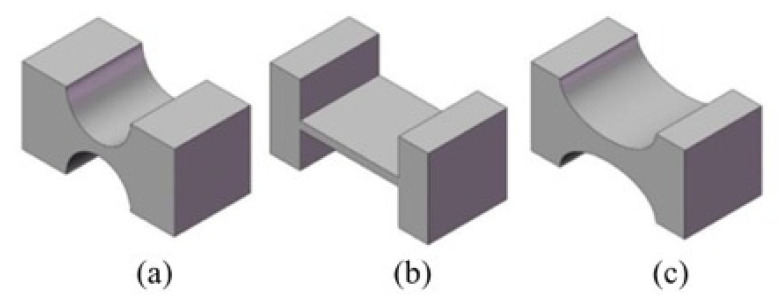
Three common notched flexure hinges: (**a**) Straight-circular flexure hinge; (**b**) straight-beam flexure hinge; (**c**) elliptical flexure hinge.

For the design of the microdissection flexure hinge in our device, the flexibility of the output shaft of the microdissection device in the axial directionshould be as large as possible, and the flexibility of the output shaft of the microdissection device in the lateral directions should be as small as possible [23]. Several common notched flexure hinges are shown in Figure 2. They are straight-circular flexure hinges, straight-beam flexure hinges, and elliptical flexure hinges. The straight-circular flexure hinge has better out-of-plane rigidity, but the displacement range is small. The straight-beam flexure hinge has a larger displacement range, but the transmission accuracy and outer surface rigidity are poor. The elliptical flexure hinge has a good displacement range and rigidity, but its design calculation and process are complicated. Considering that the object of microdissection is paraffin-embedded pathological tissue slices and the axial dissecting displacement is small, the straight-circular flexure hinge is selected as the basic unit of the flexure mechanism for design and analysis.

**Figure 3:**
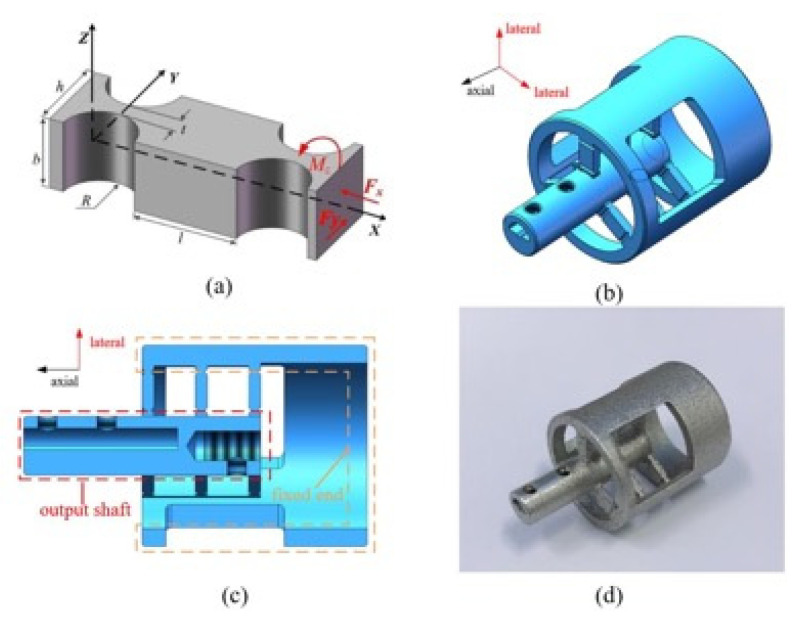
Optimized design and production process of flexure mechanism: (**a**) Schematic diagram of flexure hinge; (**b**) isometric drawing of flexure mechanism; (**c**) sectional view of flexure mechanism; (**d**) physical picture of flexure mechanism.

The flexure beam suitable for microdissection is composed of two straight-circular-flexure hinges. During the microdissection process, it is mainly subjected to two forces. Figure 3a is a simplified diagram of the force analysis of a flexure beam.

First, the output shaft of the microdissection device performs linear reciprocating high-frequency vibration along the Y-direction. This movement makes the flexure hinge beam joint bear the force $F_{y}$ and the moment $M_{z}$, causing it to undergo a certain angular deformation $\alpha_{z}$ around the Z direction and along Y. The direction is linearly deformed by $\Delta_{y}$.

Second, the weak lateral vibration generated by the axial vibration causes the flexure hinge to stretch and squeeze along the X-direction. The flexure hinge is subjected to a force $F_{x}$ and produces a linear deformation $\Delta_{x}$ along the X-direction.

Therefore, the indexes that affect the main performance of the flexure hinge are as follows: 1. The stiffness value of $M_{z}$ to generate angular deformation along the Z direction under the action of $M_{z}$, 2. Under the action of $F_{y}$, the flexibility value $\frac{\alpha_{z}}{F_{y}}$ of the angular deformation $\alpha_{z}$ in the Z direction, 3. The stiffness value $M_{z}$ of linear deformation $\Delta_{y}$ along the Y direction under the action of $M_{z}$, 4. The flexibility value $F_{y}$ of the linear deformation $\Delta_{y}$ along the Y direction under the action of $F_{y}$, 5. The flexibility value $F_{x}$ produces linear deformation along the X direction under the action of $F_{x}$.

The rotational stiffness formula of flexure hinges given by J.M. Paros [24]:

$$K=\frac{2Eb\sqrt[{5}]{t^{2}}}{9\pi \sqrt{R}}$$

The corner stiffness value is related to the hinge radius *R* and the minimum hinge distance *t*. When *R* is determined, *K* increases as *t* increases. Conversely, when *t* is determined, *K* decreases as *R* increases. To improve the axial output accuracy and resolution of the flexure mechanism, $\frac{\alpha_{z}}{M_{z}}$, $\frac{\alpha_{z}}{F_{y}}$, $\frac{\Delta y}{M_{z}}$, and $\frac{\Delta y}{F_{y}}$ should be as large as possible. At this point, R’s value should be appropriately increased, and the value of *t* should be reduced. To improve the anti-interference ability of the flexure mechanism against lateral vibration, $\frac{\Delta_{x}}{F_{x}}$ should be as large as possible. At this time, R’s value should be reduced, and the value of *t* should be increased as much as possible. Also, increasing the width b of the flexure hinge increases the overall rigidity of the flexure mechanism. Therefore, when designing the flexure hinge beam’s size, various parameters need to be adjusted repeatedly.

### 2.3. Overall Design of the Flexure Mechanism

According to the previous section analysis, the overall design of the flexure mechanism is carried out. The flexure hinge is arranged in three dimensions to avoid parasitic movement of the flexure hinge during the deformation process. Through constant size modification and simulation verification, the structure size of the flexure mechanism is finally determined. Table 1 presents the parameters of the flexure hinge.

The flexure hinge beams are distributed in a circular array at an angle of 120° with the output shaft as the axis, and three flexure hinges are distributed equidistantly on each column. Figure 3b,c show the three-dimensional model of the flexure mechanism. The flexure mechanism is divided into two parts: a fixed end and a movable end. The flexure guide mechanism’s outer casing is connected with the microdissection device to form a fixed frame. The piezoelectric ceramic output shaft and the flexure mechanism’s output shaft are fixed by a screw connection to form a movable end. To avoid the displacement of the thread gap between the output shaft of the piezoelectric actuator and the pivot shaft in the flexure mechanism, a set screw is used for reinforcement at the connection. The flexible mechanism is composed of nine flexible beams. It is a three-dimensional structure with a small volume and a complex space, and using traditional machining methods is very difficult. Therefore, the flexible mechanism is processed by 3D printing. The material is selected from 316L stainless steel with higher yield strength. The finished flexible guiding mechanism is shown in Figure 3d.

## 3. Simulation Analysis

To verify the flexure mechanism’s suppression effect on the lateral vibration, a finite element simulation comparison between the microdissection device with or without the flexure mechanism was carried out. Ignoring the threaded fit between the parts of the microdissection device, the packaged piezoelectric ceramic is regarded as a whole, and only the shaft end outputs vibration. The material properties of the microdissection device are shown in Table 2.

### 3.1. Modal Analysis

Since the microdissection device generates energy through high-frequency vibration to dissect tissue, it is necessary to perform modal analysis on the entire mechanism before selecting dissecting parameters to avoid resonance with the flexure mechanism or the entire microdissection device. Table 3 and Table 4 show the microdissection device’s resonant frequency with a flexure mechanism and the traditional microdissection device within the operating frequency range of 20 kHz–30 kHz, respectively.

### 3.2. Harmonic Response Analysis

**Figure 4:**
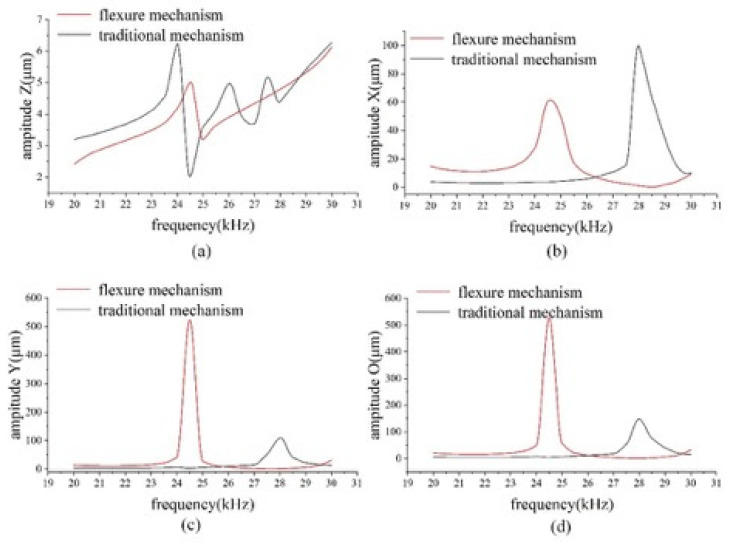
Harmonic response analysis of the needle tip vibration of the microdissection device: (**a**) The frequency response curve of the vibration amplitude in the axial Z direction of the two models; (**b**) the frequency response curve of the vibration amplitude in the lateral X direction of the two models; (**c**) frequency response curve of the vibration amplitude in the lateral Y direction of the two models; (**d**) the frequency response curve of the overall vibration amplitude of the two models.

Harmonic response analysis was carried out to obtain the displacement of the tip of the two dissection devices under high-frequency vibration. A simple harmonic dynamic load of 2 μm in the axial direction and 1 μm in the lateral direction was applied to the microdissection device with a flexural mechanism and the traditional microdissection device, respectively. The amplitude of the displacement of the two microdissection devices varies with frequency, as shown in Figure 4.

Figure 4a shows the variation curves of axial vibration amplitude with a frequency of two kinds of microdissection devices. As shown in Figure 4a, the traditional microdissection device’s axial output displacement varies significantly with the frequency and is nonlinear within the operating frequency range. In this case, the traditional dissecting needle tip’s amplitude increases unavoidably, and the experimental equipment can also be damaged easily. Therefore, it should be avoided as far as possible. The microdissection device based on the flexure mechanism has a particular amplitude jump at 24.5 kHz and 25.5 kHz, while the device can maintain good linearity at other frequencies.

Figure 4b shows the variation curve of lateral vibration amplitude and frequency on the X-direction component of two kinds of microdissection devices. As seen from the Figure 4b, the lateral vibration of the traditional microdissection device’s needle tip in the range of 20–26.5 kHz is smaller than that of the microdissection device with the flexure mechanism. In the range of 26.5–30 kHz, the microdissection device’s lateral vibration amplitude with the flexure mechanism is smaller than that of the microdissection device with the nonflexure mechanism.

Figure 4c shows the variation curve of the lateral vibration amplitude of the two microdissection devices in the Y-direction component with frequency. The variation law is similar to that of the previous Figure 4b. The needle tip’s lateral vibration in the range of 20–26 kHz of the traditional microdissection device is smaller than that of the microdissection device with a flexural mechanism. In the range of 26.5–29.5 kHz, the microdissection device’s lateral vibration amplitude with the flexure mechanism is smaller than that of the microdissection device with the nonflexure mechanism.

Lateral vibration is a physical quantity in a space, $A_{o}=\sqrt{\;A_{x}{}^{2}\;+\;A_{y}^{2}}$ is defined as the value of lateral vibration in space, where $A_{x}$ is the lateral vibration amplitude of the dissecting needle in the X-direction component, and $A_{y}$ is the lateral vibration amplitude of the dissecting needle in the Y-direction component.

Figure 4d shows the variation curve of the lateral vibration amplitude with frequency in the space of two types of microdissection devices. As shown in the figure, when the microdissection device based on the flexure mechanism works in the range of 26–29.5 kHz, its lateral vibration is significantly smaller than that of the traditional microdissection device.

**Figure 5:**
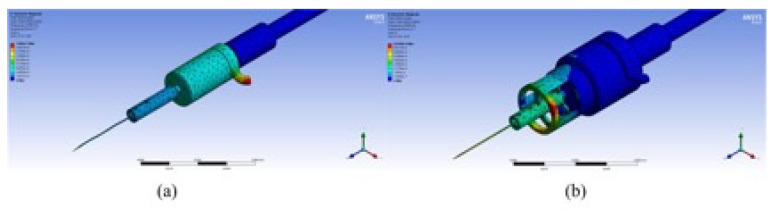
Deformation nephogram under optimal parameters of the two devices: (**a**) Deformation nephogram of the microdissection device with flexure mechanism under optimal vibration conditions; (**b**) deformation nephogram of traditional structure microdissection device under optimal vibration conditions.

In the entire working frequency range, the microdissection device’s minimum lateral vibration amplitude with the flexure mechanism is much smaller than that of the traditional mechanism microdissection device. The minimum value of the microdissection device’s lateral vibration with a flexure mechanism appears at approximately 28 kHz. Under the circumstances $A_{x}\;=\;1.13\;\mu m$ and $A_{y}\;=\;1.12\;\mu m$, the evaluation value of lateral vibration in space is $A_{o}$ = 1.59 μm. Analogously, the minimum value of the traditional microdissection device appears at approximately 22 kHz, $A_{x}\;=\;3.11\;\mu m$, $A_{y}\;=\;4.03\;\mu m$, and the evaluation value of the lateral vibration in space is $A_{o}\;=\;5.09\;\mu m$. Figure 5 shows the deformation cloud diagrams of the two microdissection devices under optimal vibration conditions.

Through the above analysis, it can be seen that the flexure mechanism dissection device has higher dissecting precision, which is conducive to the realization of high-precision microdissection. The optimal dissecting performance of the microdissection device based on the flexure mechanism is better than that of the traditional microdissection device.

## 4. Experiment and Discussion

### 4.1. Flexure Mechanism Vibration Test

To study the suppression effect of the flexure mechanism on the lateral vibration, a laser vibration measurement comparison experiment was carried out on the traditional microdissection device’s output shaft and the microdissection device with a flexure mechanism.

**Figure 6:**
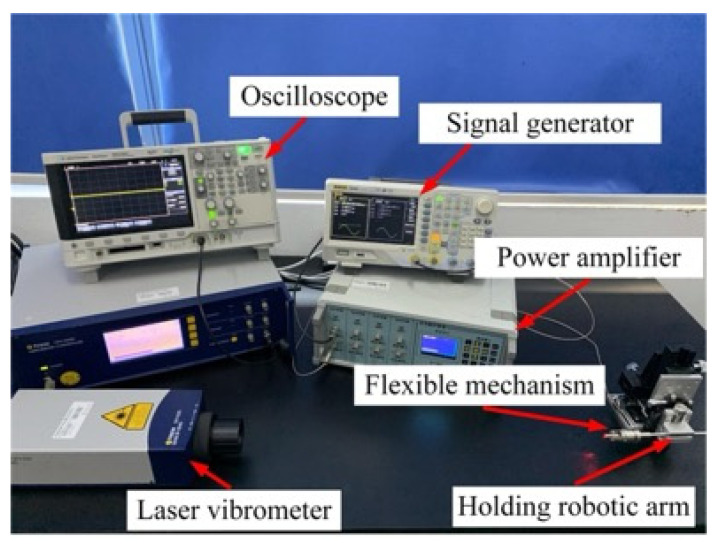
Vibration test platform for the output shaft of the microdissection device with flexure mechanism.

The experimental platform is shown in Figure 6. The test platform is composed of a laser Doppler Vibrometer (OFV-525, OFV-5000, Polytec, Karlsruhe, Germany), signal generator (DG4062, Agilent Technologies Inc, California, America), piezoelectric power amplifier (RHVD, Harbin Soluble Core Technology Co, Harbin, China), and oscilloscope (DSO-X 2022A, RIGOL Technologies Inc, Soochow, China). The laser light emitted by the vibrometer is reflected back through the dissecting needle’s output shaft, and the vibrometer converts the reflected light signal into an electrical signal, which is finally displayed on the oscilloscope. Through data analysis, the electrical signal is finally converted into the displacement information of the output shaft.

**Figure 7:**
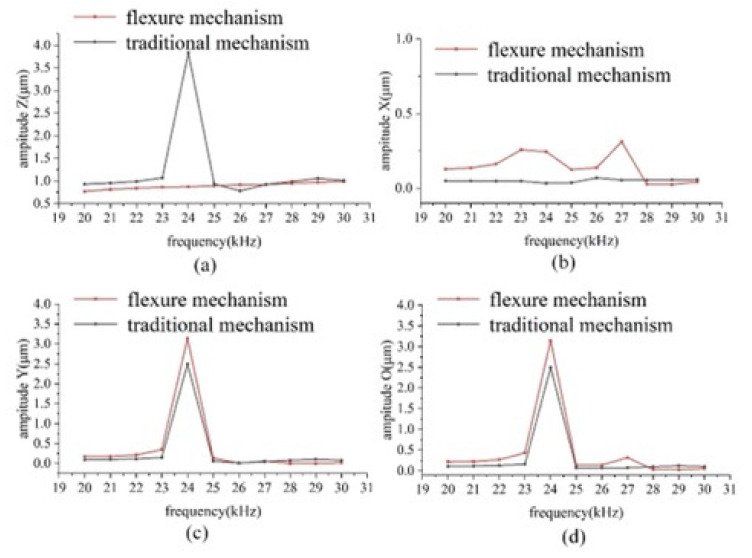
Vibration test of the output shaft of the microdissection device: (**a**) The frequency response curve of the vibration amplitude in the axial Z direction of the output shaft of the two structures; (**b**) the frequency response curve of the vibration amplitude in the lateral X direction of the output shaft of the two structures; (**c**) the frequency response curve of the vibration amplitude in the lateral Y direction of the output shaft of the two structures; (**d**) the frequency response curve of the vibration amplitude of the output shaft of the two structures in space.

A driving voltage of 8 V was applied to the piezoelectric ceramics to test its response between 20 kHz and 30 kHz. The test results are shown in Figure 7.

Figure 7 shows that the vibration amplitude of the traditional microdissection device’s output shaft fluctuates wildly, and the output shaft of the microdissection device with a flexural mechanism is linear. Under lower frequency excitation (20–28 kHz), the traditional microdissection device structure has a smaller lateral amplitude than the microdissection device with a flexure mechanism. When the driving frequency reaches 28–30 kHz, the lateral vibration amplitude of the output shaft of the microdissection device with the flexure mechanism is smaller than that of the traditional structure microdissection device.

In the working frequency range, the minimum lateral vibration amplitude of the output shaft of the new device is much smaller than that of the traditional device. Therefore, the introduction of the flexure mechanism has a positive meaning for improving microdissection performance. The optimal vibration conditions of the two structures are shown in Table 5.

### 4.2. Dissecting Needle Vibration Test

**Figure 8:**
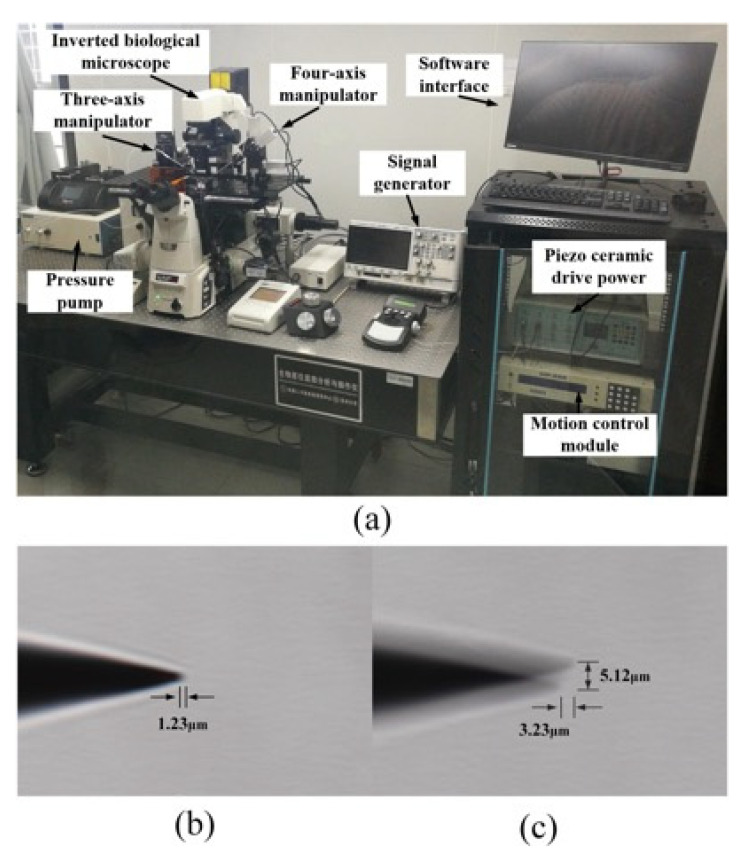
Dissecting needle tip vibration test: (**a**) The experiment platform; (**b**) the vibration of the microdissection device with flexure mechanism when the lateral vibration is minimal; (**c**) the vibration of traditional microdissection device when lateral vibration is minimum.

Since the dissecting needle tip diameter is only 1 μm, it is difficult for laser vibrometers to measure the vibration of a needle tip. Therefore, the dissecting tool tip’s actual vibration can be determined by observing the residual image of the dissecting tool tip vibration under the microscope. The experimental platform shown in Figure 8 was established. The microdissection device is installed on the high-precision manipulator on the left side of the micromanipulation experiment platform, and the angle with the horizontal plane is 30°.

A 10 V sinusoidal excitation voltage is applied to the two microdissection devices’ piezoelectric ceramics, and their vibration is tested within 20–30 kHz.

The stable lateral amplitude of the traditional microdissection device’s tip in the test frequency range is 4.87–8.96 μm. The minimum lateral vibration occurs at approximately 22.5 kHz. At this time, the axial vibration is approximately 3.23 μm, and the lateral vibration is approximately 4.87 μm.

The microdissection device with a flexure mechanism designed in this paper has a stable lateral amplitude of 0~6.76 μm in the test frequency range, and the minimum lateral vibration occurs near 28 kHz. At this time, the axial vibration amplitude is approximately 1.23 μm, and there is almost no lateral vibration. It is challenging to observe the magnitude of the lateral vibration through a microscope. Figure 8b,c shows the vibration of the two microdissection devices under a microscope.

It can be seen from the experimental results that the flexure mechanism has a specific suppression effect on both the lateral vibration and axial vibration of the needle tip. Under the same driving voltage, the microdissection based on the flexure mechanism has a smaller transverse vibration amplitude. Therefore, microdissection based on the flexure mechanism has better dissecting precision.

### 4.3. Paraffin Tissue Slice Dissecting Experiment

**Figure 9:**
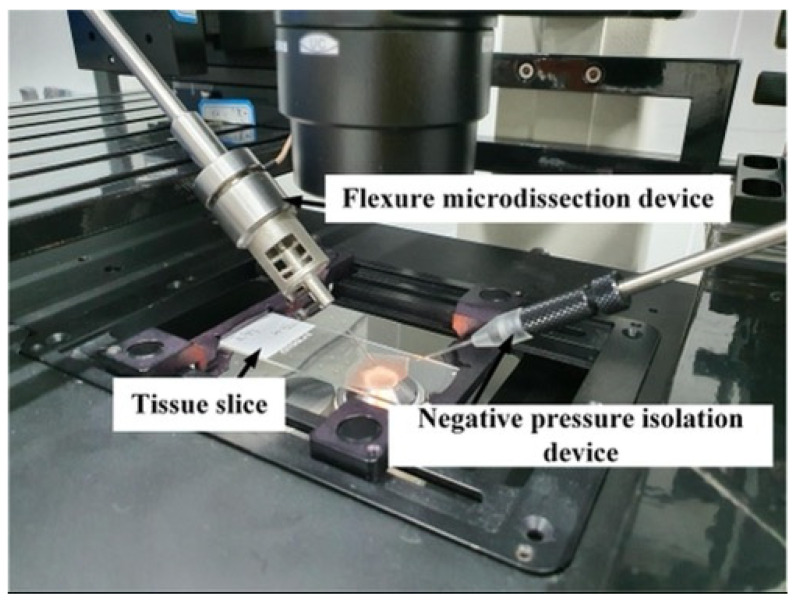
Experimental platform for tissue section dissection.

To verify the designed microdissection device’s actual performance, we conducted dissection experiments on paraffin tissue sections. First, we studied the dissecting parameters of 5 μm thick rat liver slices, and the best dissecting parameters were obtained. The main factors affecting the dissecting quality are the dissecting methods, the driving voltages, the feed speeds, the dissecting frequency, and the dissecting angles. Figure 9 shows the experimental platform for tissue section dissection.

**Figure 10:**
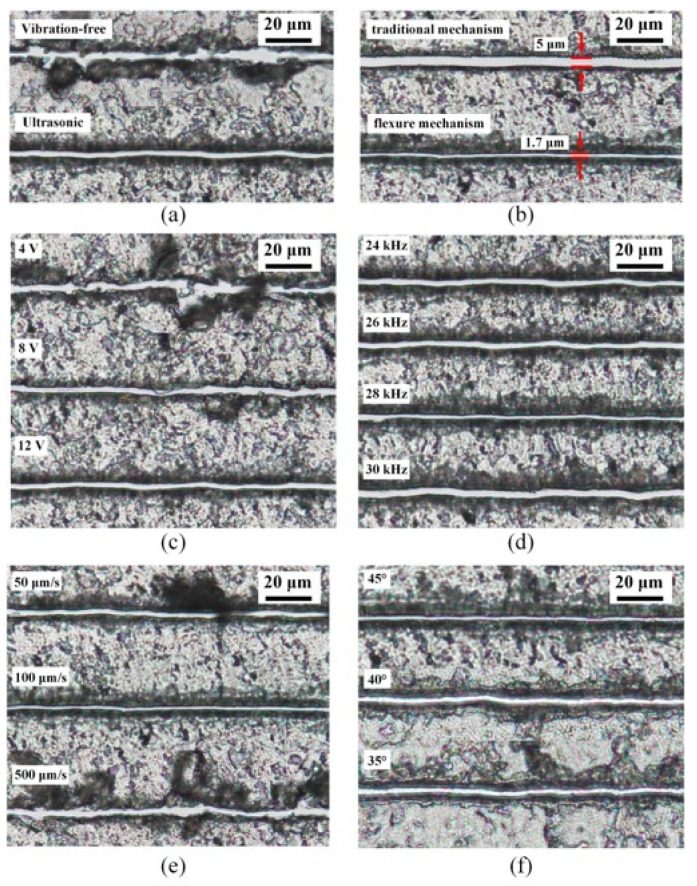
Experimental results of tissue section dissection under different dissecting parameters: (**a**) different dissecting methods; (**b**) dissecting effect of two structures; (**c**) different drive voltages; (**d**) different drive frequencies; (**e**) different feed speeds; (**f**) different dissecting angles.

First, vibration-free dissection and ultrasonic dissection was used to dissect the tissue slices. The dissecting effect is shown in Figure 10a. By observing the two dissecting results, we can find that the dissecting track’s width obtained by adopting the vibration-free mechanical dissecting method is not uniform. There is a local tissue peeling phenomenon, accompanied by a large number of chips and folds, so the dissecting effect is poor. In contrast, the path dissected by ultrasonic vibration is smoother and more completely separated from the surrounding tissue.

Next, the ultrasonic vibration dissecting method was used to dissect the tissue slices. The main factors affecting the dissecting effect include dissecting angle, driving voltage, feed speed, and driving frequency.

When the driving sinusoidal voltage on piezoelectric ceramics is 4 V, 8 V, and 12 V, the dissecting effect is shown in Figure 10c. From the experimental results, we can see that when the driving voltage is small, the energy generated by ultrasonic vibration is small, so the dissecting track presents a similar effect to mechanical dissection. As the driving voltage increases, the dissecting effect gradually improves. However, when the driving voltage is too large, a larger lateral amplitude is generated, which will not only affect the dissecting effect but also accelerate the wear of the dissecting needle. Therefore, it is not advisable to use excessive driving voltage.

The feed speed of the dissecting needle is also a key factor affecting the quality of ultrasonic dissection. Feed speeds of 50 μm/s, 100  μm/s, and 500  μm/s were used to dissect the slices for comparison experiments. The experimental results are shown in Figure 10d. When the feed speed is slow, the chips tend to adhere to the tip, thus affecting the dissecting track’s precision. Moreover, the chips may fall off during dissection, affecting the subsequent collection work. When the feed speed is too fast, the contact time between the dissecting needle and the tissue is short. The tissue cannot be separated by sufficient ultrasonic energy, so the dissecting effect is similar to that of mechanical dissecting.

The influence of frequency on the dissecting effect is mainly reflected in the dissecting precision. When the appropriate driving voltage, feed speed, and dissecting angle are selected, different driving frequencies can achieve a good tissue separation effect. Dissecting experiments were conducted at drive frequencies of 24 kHz, 26 kHz, 28 kHz, and 30 kHz for tissue slices, as shown in Figure 10d. As seen from the experimental results, when the microdissection device works at 28 kHz, the lateral vibration is the smallest, and the trajectory is the smallest, which is conducive to the realization of precise tissue dissection.

The dissecting angle is also one of the main factors that affect the dissecting effect. The liver slices were dissected at 45°, 40°, and 35°. The experimental results are shown in Figure 10e. When the dissecting needle and the dissected tissue were at an angle of 45°, the dissecting track was clear, the residual tissue was evenly divided, and the dissecting effect was the best.

The best dissecting parameters for 5 μm thick rat liver slices were finally obtained through repeated experimental tests. The best dissecting effect can be obtained when the system’s operating frequency is 28 kHz, the driving voltage is 12 V, the feed speed is 100 μm/s, and the dissecting angle is 45°.

The microdissection device with the traditional mechanism and the microdissection device with the flexure mechanism are compared. Through repeated experiments, the optimal dissecting parameters of the microdissection device with the traditional mechanism are obtained. The best dissecting effect can be obtained when the microdissection device with the traditional mechanism works at 22 kHz, the driving voltage is 12 V, the feed speed is 100 μm/s, and the dissecting angle is 45°. The comparative experimental results of the microdissection device’s optimal dissecting parameters with the traditional mechanism and the microdissection device with the flexure mechanism are shown in Figure 10b. We can clearly see that the large lateral vibration generated by the traditional dissection device is evident on the dissecting track. Jagged marks exist at the edge of the track, and tissue residues caused by lateral vibration remain on the dissecting track. In contrast, the microdissection device with a flexure mechanism has a finer track, cleaner separation, and better dissecting effect.

**Figure 11:**
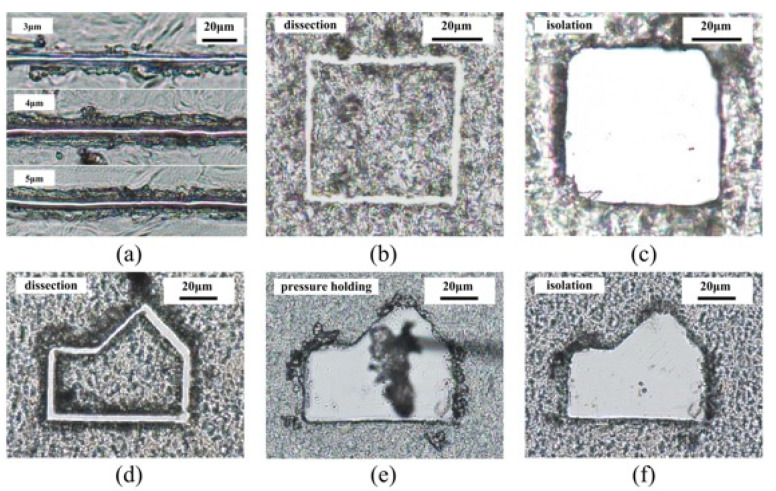
Experimental results of microdissection of different thicknesses and different tissues: (**a**) Different thickness of rat bone tissue sections; (**b**) square area dissecting of rat lung slices; (**c**) isolation of dissecting area of rat lung slices; (**d**) polygonal area dissecting of rat liver slices; (**e**) negative pressure holding isolated tissue; (**f**) isolation of dissecting area of rat liver slices.

Under the optimal microdissection parameters, dissection and isolation experiments were carried out on different thicknesses and different types of paraffin tissue sections. Figure 11a shows the microdissection effects on rat bone tissue sections of different thicknesses. It can be seen from the experimental results that the new device has good dissection performance on 3 μm, 4 μm, and 5 μm paraffin sections. Moreover, the dissecting track is clear and smooth. As the slice thickness increases, the remaining tissue at the edge of the dissecting track gradually increases. When the thickness is greater than 5 μm, the dissecting effect becomes poor. The main reason is that the flexible mechanism suppresses not only harmful lateral vibration but also restricts axial vibration. Therefore, increasing the axial vibration energy is the key to achieving thicker tissue dissection. Figure 11b,c show the dissection results and isolation of 5 μm thick rat lung tissue sections. After dissection of a square area of rat lung tissue, the area was collected and isolated by a vacuum negative pressure device. After isolation, there was no residual tissue in the centerslice’s center, and the isolation effect was fine. To show the cutting effect in different directions, we also conducted dissecting experiments on complex polygonal areas. Figure 11d–f show the experimental results of dissecting, negative pressure holding, and isolating rat liver slices’ polygonal area. Due to the adhesion between the tissue and the glass substrate, the negative pressure holding device needs to repeatedly hold the cutting area to separate the tissue. From the experimental results, the newly designed dissecting device can realize cutting tasks in different directions. In Figure 10 and Figure 11, we dissected three different kinds of tissue slices. There is no apparent difference in dissecting effect. The reason may be that in the process of making tissue sections, paraffin wax has become the main component. In the process of ultrasonic vibration microdissection, the contact time between the dissecting needle and the tissue is extremely short. When the tissue ruptures, the dissecting needle is no longer in close contact with the dissection, making ultrasonic energy difficult to transmit. Therefore, ultrasonic microdissection is less likely to cause damage to tissue DNA, RNA, and protein.

**Figure 12:**
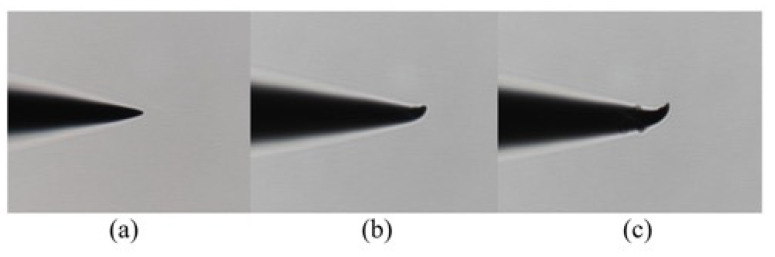
Experimental results of the dissecting needle tip wear: (**a**) Unused needle tip; (**b**) wear condition of tip of the microdissection device with flexure mechanism; (**c**) wear condition of tip of the microdissection device with the traditional mechanism.

Excessive lateral vibration also aggravates the wear of the dissecting needle and worsen the dissecting effect. After 30 identical dissecting tasks, the wear of the two dissecting tips was observed. Figure 12 shows an observation picture of the tip deformation under a microscope. It can be seen from the figure that the tip deformation of the microdissection device with the traditional mechanism is severe. Because the flexure mechanism has a good suppressing effect on the lateral vibration, the tip wear is less during dissection.

## 5. Conclusions

This paper proposes a high-precision piezoelectric ultrasonic microdissection device based on a novel flexural mechanism. Through a flexure hinge design and simulation optimization, the dissection device’s structural design parameters are determined. The newly designed microdissection device can significantly reduce the harmful lateral vibration and make the tip vibration more stable, which is proven by simulation and experiment. The influence of driving voltage, feed speed, dissecting frequency, and dissecting angle on the dissecting effect was investigated through experimental analysis of paraffin sections of rat liver with a thickness of 5 μm. Finally, after repeated experimental analysis, the optimal dissecting parameters are determined. We conducted various tissue dissection experiments to test the performance of the newly designed device, including three kinds of mouse tissue paraffin sections (liver, lung, bone) dissection experiments, different thickness tissue sections (3 μm, 4 μm, 5 μm) dissection experiments, and complete tissue sampling and extraction experiments. The results prove that our newly designed dissecting device has better dissecting precision and dissecting effect and improves the service life and durability of the dissecting needle.

## Figures and Tables

**Table 1:** Dimensional parameters of the flexure hinge.

R (mm)	*t* (mm)	l (mm)	b (mm)	h (mm)	Flexure Number
0.2	0.6	2	2	1	9 (3 × 3)

**Table 2:** Material properties of the microdissection device with a flexure mechanism.

Components	Material	Density *ρ* (g/cm^3^)	Young’s Modulus E (GPa)	Poisson’s Ratio
Dissecting needle	Tungsten	19.35	405	0.28
Flexure mechanism	316L SS	7.89	206	0.3
Packaging Housing, Outer shell	304 SS	7.93	194.02	0.3

**Table 3:** The resonant frequency of the microdissection device with a flexure mechanism.

Modal	1	2	3
Frequency (kHz)	22.304	22.557	29.633

**Table 4:** The resonant frequency of the traditional microdissection device.

Modal	1	2	3	4	5	6	7
Frequency (kHz)	21.64	23.012	23.648	27.646	27.936	28.923	29.017

**Table 5:** The optimal vibration parameters of the output shaft of the two structure devices.

	Frequency (kHz)	*A_Z_* (μm)	*A_x_* (μm)	*A_y_* (μm)	*A_o_* (μm)
Traditional mechanism	26	0.779	0.009	0.069	0.070
Flexure mechanism	28	0.947	0.002	0.027	0.027
